# The betainic form of (imidazol-2-yl)phenylphosphinic acid hydrate

**DOI:** 10.1107/S1600536810018337

**Published:** 2010-05-22

**Authors:** Peter C. Kunz, Walter Frank

**Affiliations:** a Institut für Anorganische Chemie und Strukturchemie, Heinrich-Heine-Universität Düsseldorf, Universitätsstrasse 1, D-40225 Düsseldorf, Germany

## Abstract

Single crystals of the title compound, (imidazolium-2-yl)phenyl­phosphinate monohydrate, C_9_H_9_N_2_O_2_·H_2_O, were ob­tained from methanol/water after deprotection and oxidation of bis­(1-diethoxy­methyl­imidazol-2-yl)phenyl­phosphane. In the structure, several N–H⋯O and P—O⋯H–O hydrogen bonds are found. π–π inter­actions between the protonated imidazolyl rings [centroid–centroid distance = 3.977 (2) Å] help to establish the crystal packing. The hydrate water mol­ecule builds hydrogen bridges to three mol­ecules of the phosphinic acid by the O and both H atoms.

## Related literature

For structures of related imidazolyl phosphinic acids, see: Ball *et al.* (1984 ▶); Britten *et al.* (1993 ▶). For the chemistry of imidazolyl phosphanes, see: Enders *et al.* (2004 ▶); Kimblin *et al.* (1996*a* ▶,*b* ▶, 2000*a* ▶,*b* ▶); Kunz *et al.* (2003 ▶).
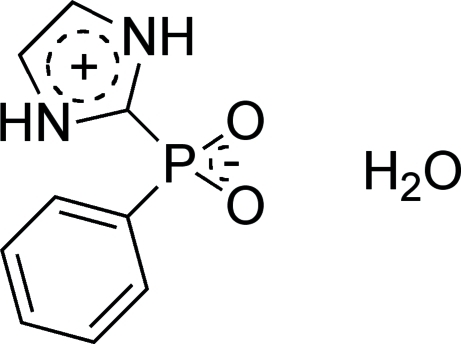


## Experimental

### 

#### Crystal data

C_9_H_9_N_2_O_2_P·H_2_O
*M* *_r_* = 226.17
Monoclinic, 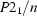

*a* = 8.5890 (6) Å
*b* = 12.1091 (7) Å
*c* = 10.9534 (7) Å
β = 111.766 (7)°
*V* = 1057.99 (12) Å^3^
*Z* = 4
Mo *K*α radiation
μ = 0.25 mm^−1^
*T* = 223 K
0.2 × 0.2 × 0.2 mm

#### Data collection

Stoe IPDS diffractometer
14882 measured reflections
2069 independent reflections
1606 reflections with *I* > 2σ(*I*)
*R*_int_ = 0.052

#### Refinement

*R*[*F* ^2^ > 2σ(*F* ^2^)] = 0.037
*wR*(*F* ^2^) = 0.092
*S* = 0.93
2069 reflections
148 parameters
H atoms treated by a mixture of independent and constrained refinement
Δρ_max_ = 0.40 e Å^−3^
Δρ_min_ = −0.16 e Å^−3^

### 

Data collection: *EXPOSE* in *IPDS Software* (Stoe & Cie, 2000 ▶); cell refinement: *CELL* in *IPDS Software*; data reduction: *INTEGRATE* in *IPDS Software*; program(s) used to solve structure: *SHELXS97* (Sheldrick, 2008 ▶); program(s) used to refine structure: *SHELXL97* (Sheldrick, 2008 ▶); molecular graphics: *SHELXTL* (Sheldrick, 2008 ▶); software used to prepare material for publication: *SHELXL97*.

## Supplementary Material

Crystal structure: contains datablocks I, global. DOI: 10.1107/S1600536810018337/nc2181sup1.cif

Structure factors: contains datablocks I. DOI: 10.1107/S1600536810018337/nc2181Isup2.hkl

Additional supplementary materials:  crystallographic information; 3D view; checkCIF report

## Figures and Tables

**Table 1:** Hydrogen-bond geometry (Å, °)

*D*—H⋯*A*	*D*—H	H⋯*A*	*D*⋯*A*	*D*—H⋯*A*
N1—H1⋯O1^i^	0.87	1.80	2.6302 (19)	160
N2—H2⋯O3^ii^	0.91 (2)	1.78 (2)	2.684 (2)	168 (2)
O3—H3⋯O2	0.83 (3)	1.94 (3)	2.773 (2)	177 (3)
O3—H4⋯O2^iii^	0.79 (3)	2.00 (3)	2.777 (2)	164 (3)

Symmetry codes: (i) 
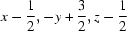
; (ii) 
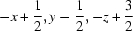
; (iii) 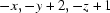.

**Fig. 1.**
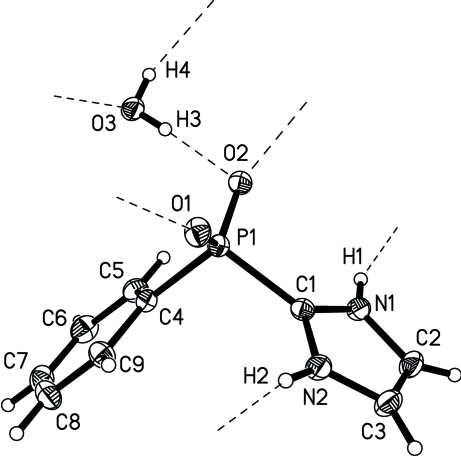
The components of the title compound with their hydrogen bond enviroment. Hydrogen atoms are drawn with an arbitrary radius and displacement ellipsoids at the 30% probability level. Dashed lines indicate hydrogen bonding establishing a three dimensional network.

**Fig. 2.**
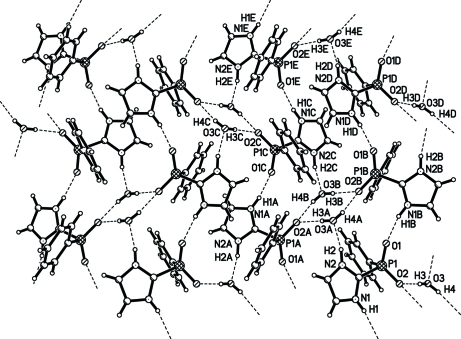
Diagram showing the supramolecular association of the betainic acid and water molecules of I in layers perpendicular to [-1 0 1]; symmetry codes: (A) x +1/2, y – 1/2, z –3/2; (B) x + 1/2, –y + 3/2, z + 1/2 (C) –x + 1, –y + 1, –z + 2; (D) x + 1, y, z + 1; (E) –x + 3/2, y – 1/2, –z + 5/2; the atoms of the unlabeled left part of the figure are generated by translation along [0 0 -1].

**Fig. 3.**
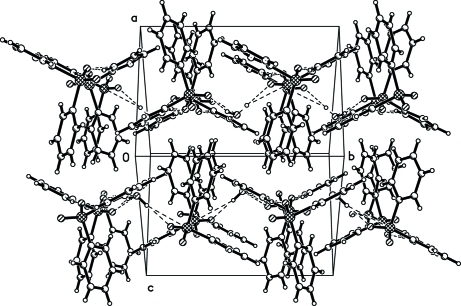
Packing diagram, view along [1 0 -1], showing the arrangement of layers perpendicular to [-1 0 1]

## supplementary crystallographic information

### Comment

Imidazolphosphanes of the type PPh_3-n_(im)_n_ (im = imidazol-2-yl) can resemble the chemistry of pyridylphosphanes PPh_3-n_(2-py)_n_ (pyridin-2-yl). In addition, imidazolylphosphanes can not only act as neutral polydentate ligands but due to their N—H functionalities act as (poly)anionic ligands, e.g. a tris(lithium) salt of tris(imidazol-2-yl)phosphane has been described (Enders *et al.*, 2004). Also, tris(imidazolyl)phosphanes and their oxides were used to mimic the tris(histidine) motif found in many metalloenzymes (Kimblin *et al.* 1996a,b, 2000a,b). The use of of imidazol-2-ylphosphanes is limited due to degradation, e.g. oxidative hydrolysis. Britten reported upon the attempted synthesis of tris(imidazol-2-yl)-phosphane. They yielded unexpectedly the bis(imidazol-2-yl)- phosphinic acid, presumably due to oxidation of the phosphane and subsequent hydrolysis (Britten *et al.*, 1993). Brown reported that the catalytic activity of a catalyst formed by zinc chloride and bis(4,5-diisopropylimidazol- 2-yl)imidazol-2-ylphosphane decreased due to formation of a zinc complex of bis(4,5-diisopropylimidazol-2-yl)phosphinic acid as degradation product (Ball *et al.*, 1984). Here we report that the oxidation of bis(imidazol-2-yl)phosphane using H_2_O_2_ in methanol did not yield the corresponding phosphane oxide, too. Upon oxidation in the presence of water, hydrolysis occurred to imidazol-2-yl phenylphosphinic acid.

The molecular structure of the title compound is shown in Figure 1. In the crystal structur of the title compound the molecules are connected into chains via N—H···O—P hydrogen bonding between the N—H H atom of the protonated imidazolyl ring and the O atom of the PO_2_ group (Figure 2 and Table 1). These chains are further connected by N—H···O and O—H···O hydrogen bonding to the water molecules. Each two water molecules and two symmetry related molecules of the title compounds forms 8-membered hydrogen bonded rings that are located on centres of inversion. Additionally to the hydrogen bonding network, in the solid state packing a pairwise π-π stacking of imidazolyl rings with a centroid distance of 3.977 Å is observed (Figure 3).

### Experimental

To a solution of 1-diethoxy-2-isopropylimidazole (Kunz *et al.*, 2003) in diethyl ether a solution of *tert.-*BuLi in hexane (1.5 M, 1.1 equivalents) is added dropwise at – 78 °C. After the solution was stirred for 30 min at – 78 °C and 30 min at room temperature the temperature is lowered again to – 78 °C and half an equivalent of dichlorophenylphosphane in diethyl ether (20 ml) is added drop-wise. After the mixture was stirred over night conc. ammonia was added, the phases were separated and the organic layer washed with bidest. water (3 x 100 ml). The organic layer was dried over anhydrous Na_2_SO_4_. After filtration and removal of the volatiles in vacuo the protected (imidazolyl)phosphane was obtained. After deprotection in an acetone-water mixture (10:1) perhydrol was added. The product precipitated as white solid which was collected by filtration, washed with acetone and diethyl ether and dried in vacuo. Single crystals were grown from a methanol / water solution. ^1^H NMR (200 MHz, [D_4_]methanol/D_2_O): δ = 7.37 (d, 2H, *J*_PH_ = 1.5 Hz, H_im_), 7.4 – 7.6 (m, 3 H, Ph), 7.85 – 8.0 (m, 2H, Ph) ppm. ^31^P{^1^H} NMR (200 MHz, [D_4_]methanol/D_2_O): δ = 3 ppm. C_9_H_9_N_2_O_2_P.H_2_O (214.16): calc. C 47.8 H 4.9 N 12.4, found C 47.2 H 4.9 N 12.1. ^1^H and ^31^P NMR spectra were recorded on a Bruker DRX 200 spectrometer. The ^1^H NMR spectra were calibrated against the residual proton signals of the solvents as internal references ([D_4_]methanol: δ = 5.84 ppm) while the ^31^P{^1^H} NMR spectra were referenced to external 85 % H_3_PO_4_.

### Refinement

Appropriate positions of all H atoms were found in difference map. The C—H atoms and the H atom of N1 were positioned with idealized geometry and refined using a riding model with U_iso_(H) = 1.2 U_eq_(C,*N*). For the O—H H atoms and the H atom at N2 positional and isotropic displacement parameters were refined.

### Crystal data

**Table:**

C_9_H_9_N_2_O_2_P·H_2_O	*F*(000) = 472
*M**_r_* = 226.17	*D*_x_ = 1.420 Mg m^−^^3^
Monoclinic, *P*2_1_/*n*	Mo *K*α radiation, λ = 0.71073 Å
*a* = 8.5890 (6) Å	Cell parameters from 8000 reflections
*b* = 12.1091 (7) Å	θ = 2.6–26.1°
*c* = 10.9534 (7) Å	µ = 0.25 mm^−^^1^
β = 111.766 (7)°	*T* = 223 K
*V* = 1057.99 (12) Å^3^	Isometric, colourless
*Z* = 4	0.2 × 0.2 × 0.2 mm

### Data collection

**Table:**

Stoe IPDS diffractometer	1606 reflections with *I* > 2σ(*I*)
Radiation source: fine-focus sealed tube	*R*_int_ = 0.052
graphite	θ_max_ = 26.1°, θ_min_ = 2.6°
Detector resolution: 6.67 pixels mm^-1^	*h* = −10→10
φ–scans	*k* = −14→14
14882 measured reflections	*l* = −13→13
2069 independent reflections	

### Refinement

**Table:**

Refinement on *F*^2^	Primary atom site location: structure-invariant direct methods
Least-squares matrix: full	Secondary atom site location: difference Fourier map
*R*[*F*^2^ > 2σ(*F*^2^)] = 0.037	Hydrogen site location: difference Fourier map
*wR*(*F*^2^) = 0.092	H atoms treated by a mixture of independent and constrained refinement
*S* = 0.93	*w* = 1/[σ^2^(*F*_o_^2^) + (0.0664*P*)^2^] where *P* = (*F*_o_^2^ + 2*F*_c_^2^)/3
2069 reflections	(Δ/σ)_max_ < 0.001
148 parameters	Δρ_max_ = 0.40 e Å^−^^3^
0 restraints	Δρ_min_ = −0.16 e Å^−^^3^

### Special details

**Table:**

Geometry. All esds (except the esd in the dihedral angle between two l.s. planes) are estimated using the full covariance matrix. The cell esds are taken into account individually in the estimation of esds in distances, angles and torsion angles; correlations between esds in cell parameters are only used when they are defined by crystal symmetry. An approximate (isotropic) treatment of cell esds is used for estimating esds involving l.s. planes.
Refinement. Refinement of *F*^2^ against ALL reflections. The weighted *R*-factor wR and goodness of fit *S* are based on *F*^2^, conventional *R*-factors *R* are based on *F*, with *F* set to zero for negative *F*^2^. The threshold expression of *F*^2^ > σ(*F*^2^) is used only for calculating *R*-factors(gt) etc. and is not relevant to the choice of reflections for refinement. *R*-factors based on *F*^2^ are statistically about twice as large as those based on *F*, and *R*- factors based on ALL data will be even larger.

### Fractional atomic coordinates and isotropic or equivalent isotropic displacement parameters (Å^2^)

**Table:**

	*x*	*y*	*z*	*U*_iso_*/*U*_eq_	
P1	0.21068 (5)	0.75986 (4)	0.66209 (4)	0.02580 (15)	
O1	0.38922 (15)	0.77558 (11)	0.74699 (13)	0.0357 (3)	
O2	0.11462 (16)	0.84520 (10)	0.56559 (13)	0.0348 (3)	
O3	−0.03925 (18)	1.01453 (13)	0.64926 (14)	0.0353 (3)	
H3	0.007 (3)	0.965 (2)	0.622 (3)	0.054 (7)*	
H4	−0.068 (3)	1.063 (3)	0.596 (3)	0.061 (8)*	
N1	0.11416 (18)	0.61686 (12)	0.44158 (15)	0.0296 (3)	
H1	0.0343	0.6596	0.3921	0.044*	
N2	0.3181 (2)	0.55296 (12)	0.60673 (16)	0.0329 (4)	
H2	0.399 (3)	0.5492 (17)	0.689 (2)	0.037 (6)*	
C1	0.2123 (2)	0.63787 (14)	0.56488 (18)	0.0269 (4)	
C2	0.1578 (3)	0.51769 (16)	0.4043 (2)	0.0383 (5)	
H10	0.1080	0.4839	0.3216	0.057*	
C3	0.2853 (3)	0.47751 (16)	0.5081 (2)	0.0403 (5)	
H11	0.3412	0.4101	0.5119	0.060*	
C4	0.0927 (2)	0.71894 (14)	0.76010 (18)	0.0276 (4)	
C5	−0.0757 (2)	0.74606 (15)	0.7218 (2)	0.0346 (4)	
H5	−0.1292	0.7856	0.6435	0.052*	
C6	−0.1654 (2)	0.71519 (18)	0.7985 (2)	0.0430 (5)	
H6	−0.2793	0.7341	0.7726	0.064*	
C7	−0.0874 (3)	0.65688 (18)	0.9125 (2)	0.0436 (5)	
H7	−0.1482	0.6361	0.9646	0.065*	
C8	0.0794 (3)	0.62866 (18)	0.9510 (2)	0.0428 (5)	
H8	0.1316	0.5883	1.0289	0.064*	
C9	0.1702 (2)	0.65964 (17)	0.87531 (19)	0.0356 (4)	
H9	0.2841	0.6406	0.9018	0.053*	

### Atomic displacement parameters (Å^2^)

**Table:**

	*U*^11^	*U*^22^	*U*^33^	*U*^12^	*U*^13^	*U*^23^
P1	0.0274 (2)	0.0255 (2)	0.0236 (3)	−0.00120 (16)	0.00835 (18)	−0.00096 (18)
O1	0.0315 (7)	0.0423 (7)	0.0312 (8)	−0.0076 (5)	0.0094 (6)	−0.0054 (6)
O2	0.0456 (7)	0.0294 (6)	0.0305 (8)	0.0057 (5)	0.0154 (6)	0.0035 (5)
O3	0.0428 (8)	0.0301 (7)	0.0295 (8)	0.0019 (6)	0.0092 (6)	−0.0007 (6)
N1	0.0308 (8)	0.0275 (7)	0.0265 (8)	0.0012 (6)	0.0060 (6)	0.0000 (6)
N2	0.0359 (8)	0.0303 (8)	0.0280 (9)	0.0054 (6)	0.0067 (7)	0.0014 (7)
C1	0.0259 (8)	0.0270 (9)	0.0263 (10)	0.0004 (6)	0.0080 (7)	0.0029 (7)
C2	0.0490 (11)	0.0325 (9)	0.0296 (10)	−0.0005 (8)	0.0104 (9)	−0.0088 (8)
C3	0.0495 (11)	0.0293 (10)	0.0380 (12)	0.0088 (8)	0.0116 (9)	−0.0036 (8)
C4	0.0310 (9)	0.0259 (8)	0.0260 (10)	−0.0017 (6)	0.0108 (7)	−0.0034 (7)
C5	0.0321 (9)	0.0321 (9)	0.0387 (11)	0.0035 (7)	0.0122 (8)	0.0000 (8)
C6	0.0351 (10)	0.0423 (11)	0.0571 (14)	−0.0004 (8)	0.0236 (10)	−0.0070 (10)
C7	0.0476 (11)	0.0486 (12)	0.0450 (13)	−0.0119 (9)	0.0291 (10)	−0.0092 (10)
C8	0.0453 (11)	0.0516 (12)	0.0316 (11)	−0.0100 (9)	0.0145 (9)	0.0042 (9)
C9	0.0317 (9)	0.0416 (10)	0.0316 (11)	−0.0012 (7)	0.0095 (8)	0.0045 (8)

### Geometric parameters (Å, °)

**Table:**

P1—O1	1.4815 (13)	C2—H10	0.9400
P1—O2	1.4897 (13)	C3—H11	0.9400
P1—C4	1.7974 (18)	C4—C5	1.388 (3)
P1—C1	1.8240 (18)	C4—C9	1.389 (3)
O3—H3	0.83 (3)	C5—C6	1.385 (3)
O3—H4	0.79 (3)	C5—H5	0.9400
N1—C1	1.325 (2)	C6—C7	1.373 (3)
N1—C2	1.365 (2)	C6—H6	0.9400
N1—H1	0.8700	C7—C8	1.377 (3)
N2—C1	1.335 (2)	C7—H7	0.9400
N2—C3	1.362 (2)	C8—C9	1.385 (3)
N2—H2	0.91 (2)	C8—H8	0.9400
C2—C3	1.344 (3)	C9—H9	0.9400
			
O1—P1—O2	121.89 (8)	C2—C3—H11	126.5
O1—P1—C4	110.00 (8)	N2—C3—H11	126.5
O2—P1—C4	109.18 (8)	C5—C4—C9	119.45 (17)
O1—P1—C1	103.90 (8)	C5—C4—P1	120.62 (14)
O2—P1—C1	105.65 (8)	C9—C4—P1	119.93 (13)
C4—P1—C1	104.68 (8)	C6—C5—C4	120.30 (19)
H3—O3—H4	109 (3)	C6—C5—H5	119.8
C1—N1—C2	109.43 (15)	C4—C5—H5	119.8
C1—N1—H1	125.3	C7—C6—C5	119.77 (18)
C2—N1—H1	125.3	C7—C6—H6	120.1
C1—N2—C3	109.27 (16)	C5—C6—H6	120.1
C1—N2—H2	123.3 (14)	C6—C7—C8	120.50 (19)
C3—N2—H2	127.4 (14)	C6—C7—H7	119.7
N1—C1—N2	107.26 (16)	C8—C7—H7	119.7
N1—C1—P1	127.65 (13)	C7—C8—C9	120.1 (2)
N2—C1—P1	125.07 (14)	C7—C8—H8	119.9
C3—C2—N1	107.04 (17)	C9—C8—H8	119.9
C3—C2—H10	126.5	C8—C9—C4	119.84 (18)
N1—C2—H10	126.5	C8—C9—H9	120.1
C2—C3—N2	106.99 (17)	C4—C9—H9	120.1
			
C2—N1—C1—N2	−0.1 (2)	O2—P1—C4—C5	14.52 (17)
C2—N1—C1—P1	178.80 (14)	C1—P1—C4—C5	−98.21 (15)
C3—N2—C1—N1	−0.1 (2)	O1—P1—C4—C9	−29.28 (17)
C3—N2—C1—P1	−179.06 (14)	O2—P1—C4—C9	−165.48 (14)
O1—P1—C1—N1	−145.99 (16)	C1—P1—C4—C9	81.80 (16)
O2—P1—C1—N1	−16.61 (18)	C9—C4—C5—C6	0.6 (3)
C4—P1—C1—N1	98.60 (17)	P1—C4—C5—C6	−179.36 (15)
O1—P1—C1—N2	32.72 (18)	C4—C5—C6—C7	−0.4 (3)
O2—P1—C1—N2	162.09 (15)	C5—C6—C7—C8	−0.1 (3)
C4—P1—C1—N2	−82.69 (17)	C6—C7—C8—C9	0.4 (3)
C1—N1—C2—C3	0.3 (2)	C7—C8—C9—C4	−0.2 (3)
N1—C2—C3—N2	−0.3 (2)	C5—C4—C9—C8	−0.3 (3)
C1—N2—C3—C2	0.3 (2)	P1—C4—C9—C8	179.68 (16)
O1—P1—C4—C5	150.71 (14)		

### Hydrogen-bond geometry (Å, °)

**Table:**

*D*—H···*A*	*D*—H	H···*A*	*D*···*A*	*D*—H···*A*
N1—H1···O1^i^	0.87	1.80	2.6302 (19)	160
N2—H2···O3^ii^	0.91 (2)	1.78 (2)	2.684 (2)	168 (2)
O3—H3···O2	0.83 (3)	1.94 (3)	2.773 (2)	177 (3)
O3—H4···O2^iii^	0.79 (3)	2.00 (3)	2.777 (2)	164 (3)

Symmetry codes: (i) *x*−1/2, −*y*+3/2, *z*−1/2; (ii) −*x*+1/2, *y*−1/2, −*z*+3/2; (iii) −*x*, −*y*+2, −*z*+1.
